# Sarcomatoid Transformation of Pulmonary Mucinous Adenocarcinoma as a Mechanism of Immunotherapy Resistance

**DOI:** 10.1111/1759-7714.70327

**Published:** 2026-06-17

**Authors:** Kensuke Yanai, Tomohiro Tanaka, Konomi Kimura, Shinji Hirose, Hiroki Watanabe, Takaaki Masuda, Ryo Suzuki, Kohei Kushiro, Naohiro Yanagimura, Shuhei Kondo, Koichiro Nozaki, Yu Saida, Miyuki Sato, Satoshi Watanabe, Hajime Umezu, Koichi Hagiwara, Toshiaki Kikuchi

**Affiliations:** ^1^ Department of Respiratory Medicine and Infectious Diseases Niigata University Medical & Dental Hospital Niigata Japan; ^2^ Division of Molecular and Diagnostic Pathology Niigata University Graduate School of Medical and Dental Sciences Niigata Japan; ^3^ Division of Pathology Niigata University Medical and Dental Hospital Niigata Japan; ^4^ Division of Pulmonary Medicine, Department of Medicine Jichi Medical University Shimotsuke Japan

**Keywords:** epithelial‐to‐mesenchymal transition, immune‐checkpoint inhibitors, invasive mucinous adenocarcinoma, sarcomatoid transformation

## Abstract

Histological transformation to sarcomatoid carcinoma is an extremely rare mechanism of resistance to tyrosine kinase inhibitors. Among adenocarcinoma subtypes, transformation of pulmonary invasive mucinous carcinoma (IMA) into other histological types has not previously been reported. We present the case of a 74‐year‐old man referred to our hospital with abnormal lung shadows. Chest computed tomography (CT) revealed extensive consolidation predominantly in the right lower lobe. Transbronchial biopsy specimens confirmed IMA, which was negative for thyroid transcription factor‐1, and a *BRAF* D594G mutation was detected. As surgical resection was not feasible, first‐line treatment with ipilimumab plus nivolumab combined with chemotherapy was initiated, resulting in a partial response. After 19 cycles of maintenance therapy, treatment was discontinued because of suspected interstitial lung disease (ILD). Thirteen months later, the patient developed radiographic worsening of ILD, for which oral prednisolone was initiated. Two months later, CT imaging revealed rapid disease progression, with enlargement of right supraclavicular, mediastinal, and abdominal lymph nodes. Re‐biopsy of a mediastinal lymph node demonstrated sarcomatoid carcinoma positive for vimentin and pan‐cytokeratin AE1/AE3. Genomic analyses again identified the *BRAF* D594G mutation, confirming sarcomatous transformation of the original IMA. The patient received two cycles of nab‐paclitaxel plus carboplatin without response and subsequently died. These findings emphasize that clinicians should recognize that IMA may undergo sarcomatous transformation.

## Graphical abstract

A patient with a pulmonary invasive mucinous adenocarcinoma (IMA) underwent a histological transformation into sarcomatoid carcinoma with multiple lymph node metastases following long‐term immunochemotherapy. *BRAF* D594G mutation was detected both at baseline and at the time of recurrence. Following the tissue transformation, vimentin became positive and E‐cadherin became negative on immunohistochemistry (IHC), suggesting that epithelial‐mesenchymal transition may play a critical role in the mechanism of sarcomatous transformation.

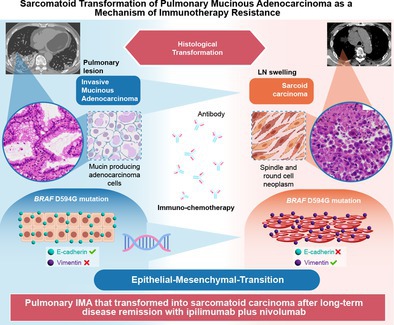

## 1 Introduction

Transformation to other histological types, such as small cell lung cancer, large cell neuroendocrine carcinoma, squamous cell carcinoma, and sarcomatoid carcinoma, has been reported as an acquired mechanism mainly in lung adenocarcinomas treated with tyrosine kinase inhibitors (TKIs) [1, 2, 3]. Among these transformations, the shift from lung adenocarcinoma to sarcomatoid carcinoma is extremely rare compared with other phenotypic conversions. Primary pulmonary invasive mucinous adenocarcinomas (IMAs), previously known as mucinous bronchioloalveolar carcinomas, represent a distinct subtype and account for approximately 3%–10% of lung adenocarcinomas [4].

Here, we report a case of pulmonary IMA that underwent histologically transformation to sarcomatoid carcinoma after immuno‐chemotherapy.

## 2 Case Presentation

**FIGURE 1:**
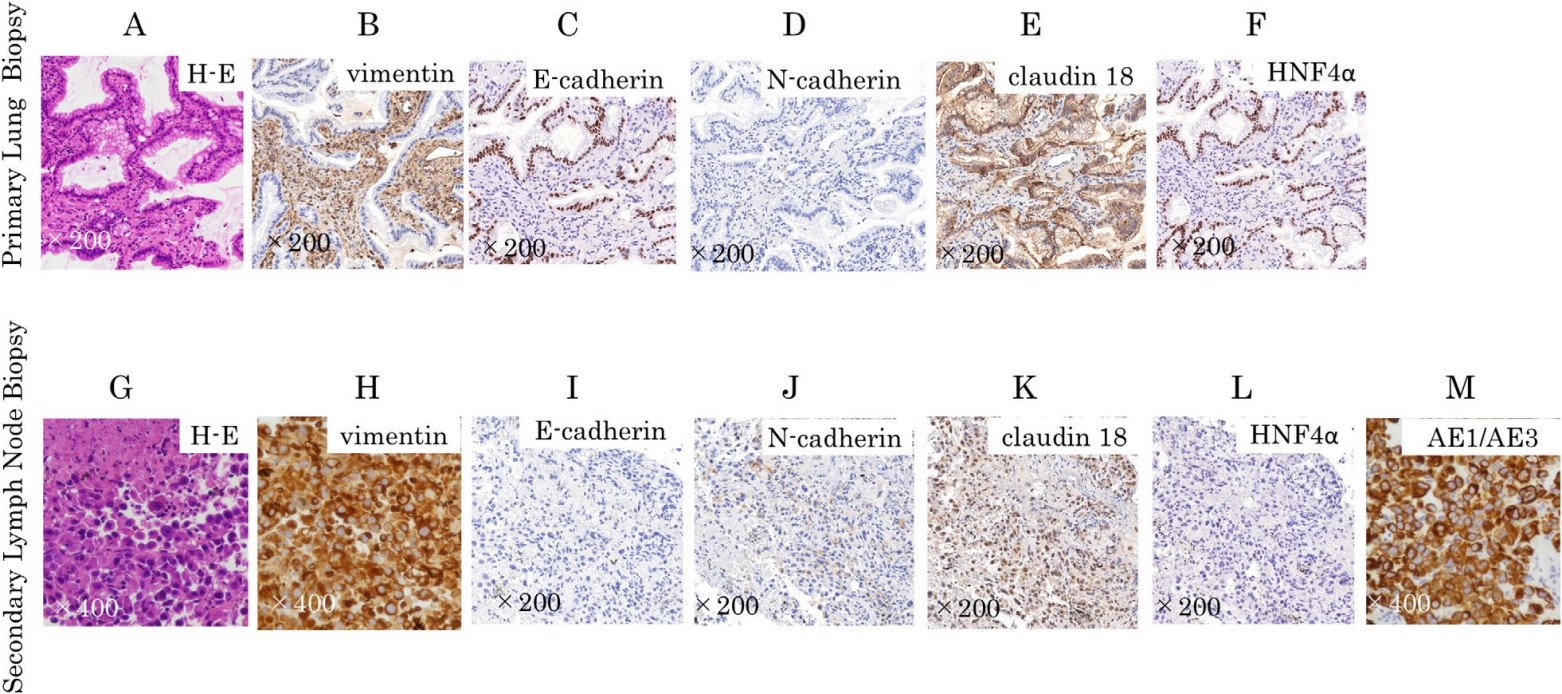
Microscopic findings of primary lung biopsy (A–F) and secondary lymph node biopsy (G–M) specimens. Hematoxylin–eosin (H–E) staining is shown in panels A and G. Immunohistochemistry staining is shown for vimentin (B and H), E‐cadherin (C and I), N‐cadherin (D and J), claudin 18 (E and K), hepatocyte nuclear factor 4α (HNF4α) (F, L), and cytokeratin AE1/AE3 (M). Original magnification ×200 (A–F and I–L) and ×400 (G, H, and M).

A 74‐year‐old Asian man with a 50‐pack‐year smoking history was referred to our hospital for a persistent dry cough. His Eastern Cooperative Oncology Group performance status was one. Computed tomography (CT) revealed extensive pneumonic consolidation in the middle and lower lobes of the right lung. Bronchoscopy was performed, and pathological examination confirmed pulmonary IMA (Figure 1A). The initial diagnosis was cT4N0M0, stage IIIA (Union for International Cancer Control classification, 8th edition) non‐small cell lung cancer (NSCLC), which was deemed unresectable because of diffuse lung involvement.

Immunohistochemistry (IHC) revealed that programmed cell death‐ligand 1 (22C3 pharmDx; Dako, Agilent Technologies, Carpinteria, USA) was negative (tumor proportion score < 1%), and showed positive staining for claudin 18 and hepatocyte nuclear factor 4α (HNF4α) (Figure 1E,F) and negative staining for thyroid transcription factor‐1 (TTF‐1) and p63. The *BRAF* D594G mutation was detected using a next‐generation sequencing–based hotspot panel test, MINtS (developed as part of the NEJ021A study [UMIN000015665] and approved for NSCLC in Japan in December 2024), identifying oncogenic driver mutations with an allele frequency of 0.005 from 10 ng DNA and RNA with a sensitivity and specificity of > 0.99 [5]. As first‐line therapy, the patient received ipilimumab plus nivolumab with two cycles of carboplatin plus pemetrexed, followed by maintenance therapy with ipilimumab plus nivolumab. A follow‐up CT scan performed 5 months after treatment initiation showed a marked reduction in the pulmonary lesion.

Following 19 cycles of maintenance therapy, a chest CT scan revealed new consolidation that did not improve with ampicillin/sulbactam. Subsequently, bronchial lavage and transbronchial biopsy were performed, revealing no malignant cells or pathogenic bacteria. Because interstitial lung disease could not be ruled out, maintenance therapy was discontinued, after which the pulmonary consolidation regressed. Thirteen months later, the patient presented with a persistent cough, and high‐resolution chest CT revealed worsening consolidation localized in the right lower lobe, predominantly distributed in the subpleural region, consistent with organizing pneumonia. This adverse event was assessed as Grade 2 according to the Common Terminology Criteria for Adverse Events version 5.0. Oral prednisolone (30 mg/day) was administered with gradual tapering, leading to symptom improvement and resolution of the opacity.

Two months later, systemic CT revealed rapid enlargement of the right supraclavicular, mediastinal, and abdominal lymph nodes (LNs). Bronchoscopy and biopsy of mediastinal LNs showed spindle‐ and round‐cell neoplasms (Figure 1G). IHC staining at recurrence revealed a focal increase in vimentin expression, decreased E‐cadherin expression, and no change in N‐cadherin expression compared to baseline, along with positivity for pan‐cytokeratin AE1/AE3 and negativity for TTF‐1 and p40, consistent with transformed sarcomatoid carcinoma (Figure 1B–F,H–M).

MINtS analysis of the recurrent LN specimens again identified the *BRAF* D594G mutation, and the *BRAF* variant allele frequencies (VAF) remained nearly stable between initial diagnosis and recurrence (21.6% vs. 28.6%). This histologic transformation was identified after the organizing pneumonia resolved following steroid treatment. These findings strongly suggest that the recurrence shares a genetic origin with the initial lesion and that the recurrent LN metastases originated from the original IMAs rather than representing a secondary primary malignancy.

**FIGURE 2:**
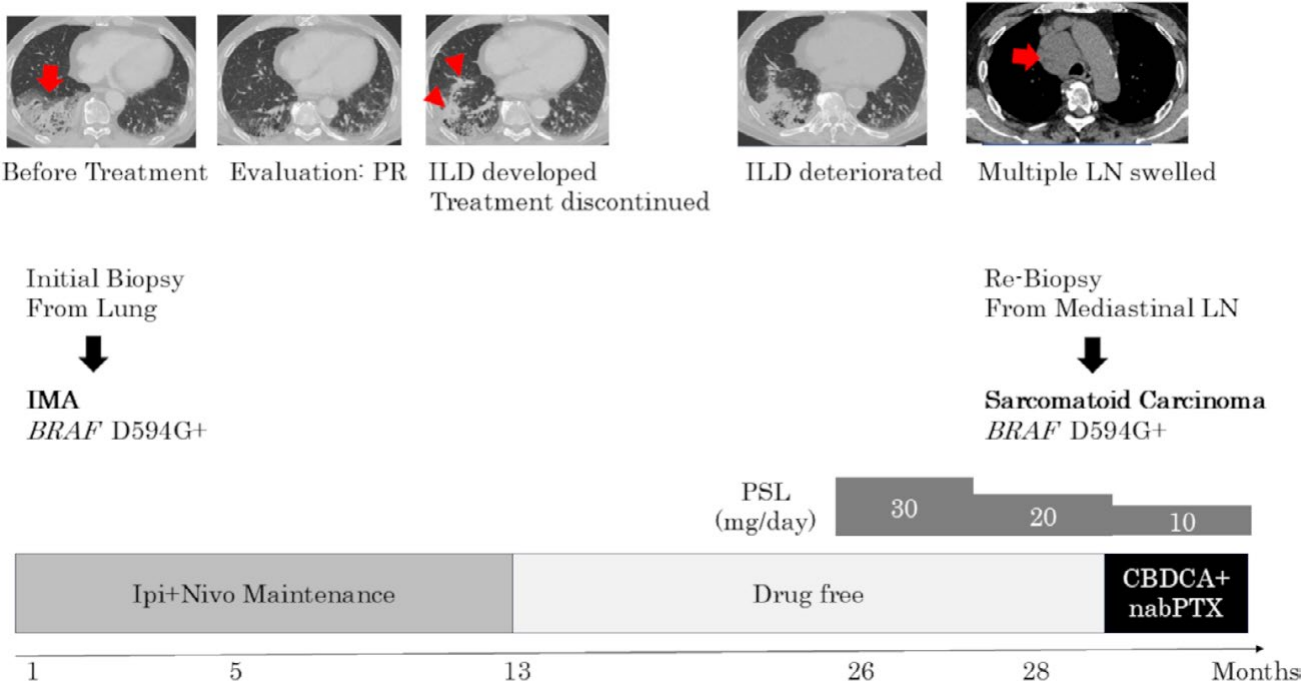
Treatment course of the patient. Abbreviations: CBDCA, carboplatin; ILD, interstitial lung disease; IMA, invasive mucinous adenocarcinoma; Ipi, ipilimumab; LN, lymph nodes; nab‐PTX, nanoparticle albumin‐bound paclitaxel; Nivo, nivolumab; OP, organizing pneumonia; PR, partial response; PSL, prednisolone.

The patient was treated with carboplatin plus nanoparticle albumin‐bound paclitaxel; however, disease progression occurred. The patient died 6 weeks after discontinuation of second‐line therapy (Figure 2).

## 3 Discussion

In this case, pulmonary IMA underwent histological transformation to sarcomatoid carcinoma without any detectable genetic alterations associated with treatment resistance after immunochemotherapy. To our knowledge, this is an extremely rare reported case of pulmonary IMA transforming into another histological type following antitumor therapy. Similar transformations to sarcomatoid carcinoma have been reported in NSCLC as mechanisms of resistance to TKIs or immunotherapy [6, 7, 8]. Previous studies have shown that survival after transformation is poor, with a median of 2.5 months (range, 1–16 months) [7].

The mechanisms underlying sarcomatoid transformation remain unclear. This process may involve epithelial‐mesenchymal transition (EMT), in which epithelial tumor cells lose polarity and cell‐to‐cell adhesion, degrade E‐cadherin, and acquire mesenchymal features such as vimentin expression [9]. Interestingly, IHC showed reduced expression of E‐cadherin, one of the EMT markers, at recurrence compared to baseline, suggesting that EMT was induced as a mechanism of acquired resistance in this patient. In the immunotherapy era, histological transformation following immune checkpoint inhibitor (ICI) therapy has also been reported in lung cancer [6]. Sarcomatoid transformation may facilitate tumor immune escape, functioning as an acquired resistance mechanism against ICI [6]. Cancer‐associated fibroblasts (CAFs) have been shown to promote EMT via paracrine signaling [10, 11]. Since the patient was diagnosed based on only a few small tissue specimens, it cannot be ruled out that the initial diagnosis represented a mixed type of mucinous and non‐mucinous adenocarcinoma, with sarcomatoid transformation arising from the non‐mucinous component. However, in the baseline tissue, only cells positive for HNF4α by IHC, specifically those of the mucinous IMA, were identified.

IMA is known for an airway‐related progression pattern, and postoperative recurrence is usually intrapulmonary; extrapulmonary metastasis occurs in only about 5% of cases [12]. In this patient, however, recurrence involved multiple LNs, suggesting that extrapulmonary spread of pulmonary IMA may serve as a clinical clue for underlying histological transformation. Therefore, if IMA possesses a lung‐specific biological pathway that induces lesion formation, it was hypothesized that a mutation within the pathway during tissue transformation could lead to the formation of extrapulmonary lesions. On the contrary, in this patient, the possibility that the tumor was a mixed‐type adenocarcinoma comprising both mucinous and non‐mucinous components (including sarcomatoid carcinoma) at the time of the initial diagnosis cannot be completely ruled out. However, IHC staining for HNF4α and claudin 18 was mainly positive in the tissue from the initial diagnosis, suggesting minimal evidence of a mixed histological pattern (Figure 1E,F).

*BRAF* is one of the *RAF* family of serine/threonine kinases, and among them, the *BRAF* D594G mutation is classified as a Class III *BRAF* (non‐V600) alteration. Class III *BRAF* mutations are reported to be low‐activity, kinase‐impaired mutations that are unresponsive to RAF inhibitors due to RAS dependency, and molecularly targeted therapy for these mutations remains under development [13]. Therefore, first‐line treatment for Class III *BRAF* mutation–positive advanced lung cancer follows the same standard therapy used for oncogenic driver mutation‐negative NSCLC, such as the immunochemotherapy administered to this patient [14]. Second‐line options for Class III *BRAF*–mutant advanced lung cancer include consideration of enrollment in clinical trials, including those investigating MEK inhibitors [13].

Currently, no standardized treatments exist for transformed sarcomatoid carcinomas. Targeted therapy may be considered when actionable mutations are present, although this is rare. Retrospective studies suggest that ICIs may be effective for *de novo* pulmonary sarcomatous carcinoma [15, 16], yet ICI rechallenge appears ineffective in cases with acquired sarcomatoid transformation [6]. Novel therapeutic strategies are urgently needed to overcome sarcomatous transformation.

A limitation of this case report is that extensive comparative genomic analysis, including passenger mutations or copy‐number alterations, was not available. Consequently, the clonal continuity before and after transformation is based solely on evidence derived from a synthesis of clinical and pathological findings, rather than on comprehensive genomic analysis.

We report a case of pulmonary IMA that transformed into sarcomatoid carcinoma after long‐term disease remission with ipilimumab plus nivolumab. Broader comparative genomic profiling was not available. Clinicians should be aware that pulmonary IMA with such involvement may indicate sarcomatoid transformation.

## Author Contributions

**Konomi Kimura:** writing – review and editing. **Shinji Hirose:** writing – review and editing. **Kensuke Yanai:** writing – review and editing. **Kohei Kushiro:** writing – review and editing. **Shuhei Kondo:** data curation, writing – review and editing. **Yu Saida:** writing – review and editing. **Takaaki Masuda:** writing – review and editing. **Tomohiro Tanaka:** writing – original draft. **Ryo Suzuki:** writing – review and editing. **Hiroki Watanabe:** writing – review and editing. **Koichiro Nozaki:** writing – review and editing. **Koichi Hagiwara:** data curation, writing – review and editing. **Naohiro Yanagimura:** writing – review and editing. **Toshiaki Kikuchi:** supervision, writing – review and editing. **Satoshi Watanabe:** writing – review and editing, supervision. **Hajime Umezu:** data curation, writing – review and editing. **Miyuki Sato:** writing – review and editing.

## Funding

This work was financially supported by Grants‐in‐Aid for Scientific Research (KAKENHI) (grant number JP300025K20595).

## Ethics Statement

Study approval was obtained from the Niigata University Institutional Review Board (IRB). This submission was approved by the patient relatives. We obtained written consent from the patient and his relatives.

## Consent

Informed consent for publishing the details of this case was obtained from the patient and his relatives.

## Conflicts of Interest

Toshiaki Kikuchi received honoraria (lecture fees) from Bristol‐Myers Squibb K.K. and Eli Lilly Japan K.K., and received grants or contracts (research fund) from Bristol‐Myers Squibb K.K.; Satoshi Watanabe received honoraria (lecture fees) from Bristol‐Myers Squibb K.K., and participated in an Advisory Board of Bristol‐Myers Squibb K.K.; Koichi Hagiwara participated in a Data Safety Monitoring and Advisory Board for EIKEN CHEMICAL CO. LTD.; Koichiro Nozaki received honoraria (lecture fees) from Bristol‐Myers Squibb K.K.; Yu Saida received honoraria (lecture fees) from Bristol‐Myers Squibb K.K. and Eli Lilly Japan K.K.; Kohei Kushiro received honoraria (lecture fees) from Eli Lilly Japan K.K.; Ryo Suzuki received honoraria (lecture fees) from Eli Lilly Japan K.K.; Tomohiro Tanaka, Kensuke Yanai, Konomi Kimura, Shinji Hirose, Hiroki Watanabe, Takaaki Masuda, Miyuki Sato, Naohiro Yanagimura, Shuhei Kondo, and Hajime Umezu declare no conflicts of interest.

## Data Availability

The original contributions presented in the study are included in the article. Further inquiries can be directed to the corresponding author.
